# Fine-scale intraspecific niche partitioning in a highly mobile, marine megafauna species: implications for ecology and conservation

**DOI:** 10.1098/rsos.221529

**Published:** 2023-06-28

**Authors:** Ian Silver-Gorges, Simona A. Ceriani, Mariana M. P. B. Fuentes

**Affiliations:** ^1^ Department of Earth, Ocean, and Atmospheric Science, Florida State University, Tallahassee, FL 32304, USA; ^2^ Fish and Wildlife Research Institute, Florida Fish and Wildlife Conservation Commission, St. Petersburg, FL 33701, USA

**Keywords:** niche partitioning, isotope, foraging, sea turtle, ecology, megafauna

## Abstract

A species may partition its realized ecological niche along bionomic and scenopoetic axes due to intraspecific competition for limited resources. How partitioning manifests depends on resource needs and availability by and for the partitioning groups. Here we demonstrate the utility of analysing short- and long-term stable carbon and nitrogen isotope ratios from imperiled marine megafauna to characterize realized niche partitioning in these species. We captured 113 loggerhead sea turtles (*Caretta caretta*) at a high-use area in the eastern Big Bend, Florida, between 2016 and 2022, comprising 53 subadults, 10 adult males and 50 adult females. We calculated trophic niche metrics using established and novel methods, and constructed Bayesian ellipses and hulls, to characterize loggerhead isotopic niches. These analyses indicated that loggerheads partition their realized ecological niche by lifestage, potentially along both bionomic (e.g. trophic) and/or scenopoetic (e.g. habitat, latitude or longitude) axes, and display different characteristics of resource use within their niches. Analysis of stable isotopes from tissues with different turnover rates enabled this first characterization of intraspecific niche partitioning between and within neritic lifestages in loggerhead turtles, which has direct implications for ongoing research and conservation efforts for this and other imperiled marine species.

## 1. Introduction

The concept of a species' ecological niche has become central to scientific understanding of the evolution of phenotypic variation and subsequently the natural maintenance of populations [1,2]. A species’ ecological niche may be thought of as an *n*-dimensional hypervolume constrained by scenopoetic (e.g. habitat and latitude) and bionomic (e.g. prey items) axes [3]. The ecological niche is further subdivided into ‘fundamental’ niches (i.e. the entire hypervolume that a species has the potential to occupy) and ‘realized’ niches (i.e. the hypervolume that a species actually occupies) [3]. Conspecifics may display overlapping realized ecological niches, for example by foraging on the same resources at the same locations [4] (except see [5] and works therein for both supporting and refuting examples). Any subsequent intraspecific competition for resources may have deleterious impacts at a population level [6–8]. However, competition can spur phenotypic variation in the form of ecological niche partitioning, in which some conspecifics expand into other areas of the species' fundamental niche by using discrete scenopoetic and/or bionomic resources [9–11].

The competition that leads to ecological niche partitioning is driven simultaneously by resource availability and individual resource requirements and choices [4,6,12]. Limited resource availability relative to population requirements increases pressure towards niche partitioning [6], but there must exist resource heterogeneity and sufficient abundance of resources for individuals to successfully partition [9,11,13]. Resource availability is also partially conditional upon individual resource requirements, which may vary by age, sex and size [10,12]. Juveniles may require more resources than adults due to requirements to grow and reach maturity [14–16]. Adults may exhibit periodic increases in resource requirements for reproduction [17,18], and this may further differ by sex [10,19] and body size [20–22]. Individuals within a population may also specialize to avoid competition, independent of their age, sex or size [23,24].

Characterizing realized ecological niches and identifying partitioning along demographic or character-trait axes requires quantifiable measures of scenopoetic and bionomic resource use [25]. Stable isotope ratios of scenopoetically and bionomically relevant elements, namely *δ*^13^C or *δ*^15^N, generated from tissue samples of focal organisms have emerged as workhorses of ecological niche and niche-partitioning characterization [25–28]. These ratios are a function of prey item isotope ratios [29,30], dietary proportions of prey items [31,32], trophic fractionation [33,34] and foraging location [34–36]. Stable isotope ratios comprise finite, *n*-dimensional data, which are suitable for modelling and characterizing isotopic niches [25–27]. Isotopic niche characterizations can then be used as proxies for realized niche characterizations, given that they integrate multiple scenopoetic and bionomic factors [25]. Further, isotope ratios reflect resource use across the turnover rate of the tissue from which they were generated [37]. Turnover rates range from hours (e.g. blood) to months (e.g. epidermis) to fixation in inert tissues (e.g. keratin; see [37] for a review of this phenomenon). Selection of tissues with various turnover rates for isotopic analysis allows characterization of temporal variability in resource use both within and between individuals [25,37,38]. These assets combined make stable isotope ratios particularly useful for characterizing realized ecological niches and ecological niche partitioning in species that are difficult to observe *in situ*, such as imperiled marine megafauna, which are highly mobile and spend most of their time at sea [39,40].

Extant populations of imperiled marine megafauna are reduced relative to historical populations often due in part to habitat loss and degradation [41–43]. Conservation plans for marine megafauna species often emphasize actions that directly increase population abundance (e.g. [44,45]), but it is difficult to account for downstream factors such as resource availability that influence population viability [46–48]. The same habitat loss and degradation that have led to population declines in imperiled species may act to reduce total available fundamental niche space for a species, increase intraspecific competition and possibly force some individuals or groups of individuals to partition into potentially less-optimal niche space [49,50]. Research that identifies intraspecific partitioning in imperiled species can help to characterize the ability of existing niche space to support populations of imperiled species and further provide impetus for the protection of vulnerable demographics and of niche space used by those species [39,40,51,52]. Characterizing niche space and partitioning at relevant spatio-temporal scales for marine megafauna species presents a logistical challenge, as individuals spend substantial portions of their lives as residents at often remote foraging areas [53–57]. Ecological niches and intraspecific partitioning are largely shaped by scenopoetic and bionomic resources at these locations [39,40]. As these species are highly mobile between foraging and breeding areas, they may be most accessible for sampling (i.e. for stable isotope analysis) just after undertaking seasonal or perennial migrations between foraging areas and/or breeding areas [39,40,58–60] and studies must be careful that characterizations of ecological niches and niche partitioning truly reflect processes at foraging areas [28,33,39]. Thus, a highly informative approach to characterize ecological niches and niche partitioning in marine megafauna species requires sampling multiple demographics at relevant locations (i.e. foraging areas) and generating scenopoetic and bionomic data that capture the temporal span of resource use [28,29].

**Figure 1.**
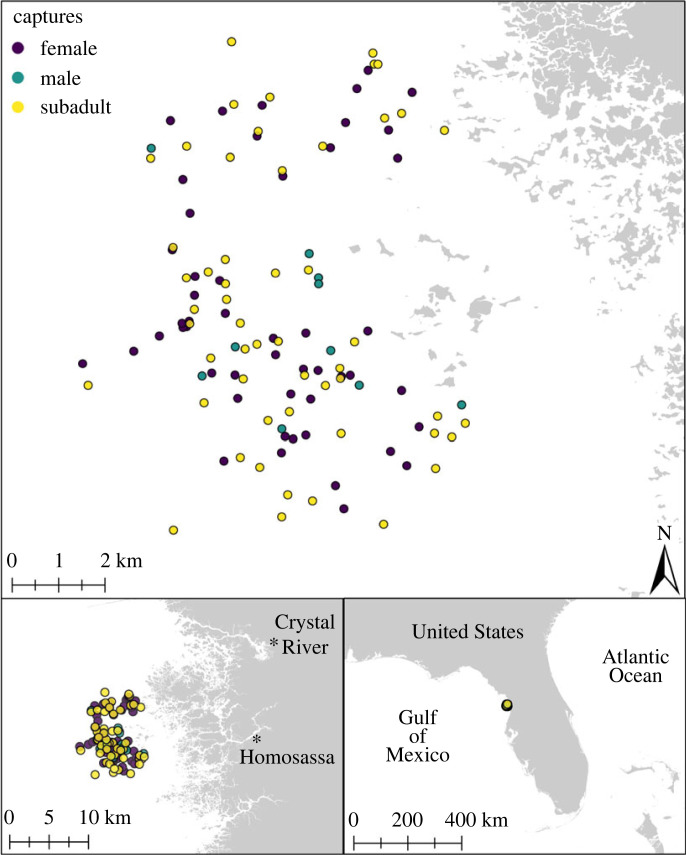
Maps of study site and loggerhead capture points at different scales. Colour indicates sex (for individuals classified as adults) or lifestage (for subadults).

We demonstrate such an approach to characterize the realized ecological niche and intraspecific niche partitioning of threatened loggerhead sea turtles (*Caretta caretta*) [61], which spend most of their lives at sea [62] and display long-term fidelity to neritic foraging areas once they recruit from oceanic developmental habitats [62,63]. Such characterizations are rare for loggerhead turtles, and for immature lifestages in sea turtles generally [64]. We analysed carbon (*δ*^13^C) and nitrogen (*δ*^15^N) isotope ratios measured from epidermis (3- to 6-month turnover rate) and scute (inert and therefore fixed [37]) samples collected from subadult and adult loggerheads encountered at a neritic foraging area in the Gulf of Mexico [65,66] (figure 1). These data were then subjected to multiple analyses to characterize the realized ecological niche of foraging loggerheads at this site, as well as to explore if ecological niche partitioning was occurring by sex, lifestage or at an individual level. In particular, different lifestages face different resource requirements (i.e. immature individuals require resources for growth, while mature individuals require resources for breeding, which varies inter- and intra-annually), and as such might exhibit different ecological niche and resource use characteristics (e.g. as in [14–18]). When put into a biological and ecological context, the results of this work exemplify how isotopic characterization of ecological niches and intraspecific niche partitioning can identify resource use strategies in, and inform conservation efforts of, sea turtles and other imperiled species globally.

## 2. Methods

Capture surveys for sea turtles were conducted in the coastal waters of the Florida Big Bend between the mouths of the Crystal and Homosassa Rivers in Citrus County, Florida (figure 1). This area has been identified as a hotspot for three sea turtle species: loggerhead, green (*Chelonia mydas*), and kemps ridley (*Lepidochelys kempii*) [65,67]. Surveys were conducted approximately bi-monthly between January 2016 and February 2022 dependent on logistics and boating conditions (see electronic supplementary material, table S1). Sea turtles were spotted opportunistically from a vessel and captured using the ‘rodeo’ method [68]. Sighting and capture GPS locations were recorded using a Garmin GPSMAP. Captured turtles were brought on board the vessel and checked for Inconel flipper tags and passive-integrated transponders (PIT). Tag numbers were recorded when already present and new flipper (National Band and Tag Company, Style 681) and PIT tags (Biomark, GPT12) were applied in the absence of old tags. Standard (nuchal notch to caudal tip) and minimum (nuchal notch to caudal notch) lengths were measured from each carapace using a tape measure (curved carapace length, CCL) and calipers (straight carapace length, SCL) [69]. Turtles sampled in-water are commonly categorized into lifestages (e.g. juvenile, subadult and adult) based on minimum curved carapace length (CCLmin) measurements [69–71]. Nesting females in the Gulf of Mexico are typically 80 cm CCLmin or larger [71], and so turtles sampled in this study were classified as either subadults (CCLmin < 80 cm) or adults (CCLmin ≥ 80 cm). This minimized, but did not eliminate, the chance of incorrectly classifying lifestages in captured turtles without performing invasive procedures such as laparoscopy (see [72] and references therein). Turtles that were classified as adults with tails that extended past their carapace were further classified as males [73]. All other turtles classified as adults were further classified as females. Subadults were not further classified by sex.

Epidermis and scute were sampled for isotopic analysis using 5 mm and 6 mm biopsy punches, respectively. Epidermis was collected from the shoulder of captured turtles after sterilization with a 95% ethanol pad and stored in dry salt (NaCl) until downstream preparation and isotopic analysis [74]. One costal scute on each carapace was selected for keratin sampling [75,76]. The selected scute was scrubbed with a Scour Pad (Scotch-Brite) and rinsed to remove small epibionts and algae. One central and one distal circular punch were made in each scute to ensure adequate sampling. Samples of all scute layers were collected, as determined by visual observation of the white carapace epithelium after removing each circular punch. Scute samples were wrapped in foil with the outer surface marked, placed in individual vials and frozen until downstream preparation and isotopic analysis. Turtles were released within 200 m of their capture locations.

Epidermis and scute samples were sent to the Fish and Wildlife Research Institute of the Florida Fish and Wildlife Conservation Commission and the Marine Environmental Chemistry Laboratory at the University of South Florida College of Marine Science for preparation and isotopic analysis. Epidermis samples were brushed and rinsed with deionized water to remove salt, separated from any underlying fat, dried in an oven for 2 h at 60°C and homogenized [74,77,78]. Lipids were extracted from epidermis and scute samples using an accelerated solvent extractor (Model 200, Dionex) with petroleum ether (three cycles of 5 min heating followed by 5 min of static purging). A 0.5–0.7 mg of each epidermis sample were weighed using a Mettler Toledo micro-balance and placed into 3 mm × 5 mm Costech tin cups. Central and distal scute samples from each turtle were measured, and the thickest scute (representing the longest time period) from each turtle was selected for analysis. The outside layer of each scute sample was glued to a glass slide and sampled in 50 µm intervals using a carbide end mill [76]. Each of these powdered scute layers was collected using a forceps with a small piece of quartz wool, to which the scute powder is electrostatically attracted. The quartz wool and associated scute powder where then wrapped in Costech aluminium foil. Skin and scute layer samples were converted to N_2_ and CO_2_ using a Carlo – Erba EA1108 Elemental Analyzer (Thermoquest Italia). Isotope ratios and percentages-by-mass were measured in a continuous flow mass spectrometer (Delta PlusXP, Thermofinnigan, Bremen). Sample ratios are expressed in per mil (‰) as calculated using the equation

$$\delta X=\left[\left(\frac{R_{\mathrm{sample}}}{R_{\mathrm{standard}}}\right)-1\right]\times 1000,$$

where *X* is ^15^N or ^13^C, and R is the ratio of ^15^N:^14^N or ^13^C:^12^C. Standards for ^15^N and ^13^C were AT-Air and VPDB, respectively. Secondary reference materials (NIST 8574 *δ*^13^C = + 37.63 ± 0.10 ‰, *δ*^15^N = + 47.57 ± 0.22 ‰, %N = 9.52%, %C = 40.81%, C:N_molar_ = 5.0; NIST 8573 *δ*^13^C = −26.39 ± 0.09 ‰, *δ*^15^N = −4.52 ± 0.12 ‰, %N = 9.52%, %C = 40.81%, C:N_molar_ = 5.0) were used to normalize raw measurements to standards. Measurements of analytical uncertainty (reflecting ± 1 s.d.) were obtained by replicate measurements (*n* = 308) of an internal laboratory reference material (NIST1577b Bovine liver, *δ*^13^C = −21.69 ± 0.14 ‰, *δ*^15^N = 7.83 ± 0.16 ‰, %N = 9.95 ± 0.48%, %C = 48.04 ± 0.71%, C:N_molar_ = 5.63 ± 0.27) and were ≤ 0.25 ‰ (*δ*^13^C) and ≤ 0.31 ‰ (*δ*^15^N).

Isotope data were imported into R v. 4.1.3 [79] for analysis. Epidermis isotope data generated in this study were compared to previously generated isotope data for loggerhead turtles foraging in the eastern Gulf of Mexico [80,81] using student's T-tests to explore variability in isotopic data between studies by individual size and spatial scale. These data included turtles that foraged across latitudes (approx. 24°−28.5°N) and depths (0–200 m), but should reflect broad trends in scenopoetic isotopic variability (discussed below) and therefore serve as an informative, if imperfect, comparisons for data from this study.

Trophic niche metrics (*sensu* [27]) were calculated to quantify within-individual variation in isotopic resource use (WIC; the extent to which individuals display more homogeneous [lower] or heterogeneous [higher] resource use over time), between individual variation in isotopic resource use (BIC; the extent to which individuals overlap [lower] or differ [higher] in resource use), isotopic niche breadth (total niche width; TNW; the span of resource use), and individual specialization (WIC/TNW; the extent to which individuals are specialists [WIC/TNW≍0] or generalists [WIC/TNW≍1] in resource use) [27,76,82–84]. These metrics were quantified for *δ*_SCUTE_ data for all turtles, as well as for adults and subadults separately to compare isotopic resource use between lifestages. These metrics are univariate, and only calculated for each isotope individually, subscripts (‘C’ for Carbon, ‘N’ for Nitrogen) are used to specify which isotope they refer to. WIC and BIC were calculated as the mean sum-of-squares within and between individuals, respectively, from ANOVAs [76,83,84]. TNW was then calculated as the sum of BIC and WIC, and WIC/TNW was calculated as the quotient of WIC divided by TNW [27,76,83,84]. ANOVAs for scute data were formulated as

δ∼layer+turtle

where *δ* represents either *δ*^13^C or *δ*^15^N, *layer* represents the layer of scute within each sample and *turtle* represents individual turtles.

These metrics are typically calculated using ANOVA frameworks [76,83,84], yet data with multiple samples from the same individual (e.g. scute isotope data) may violate assumptions of sample independence in ANOVAs. Linear mixed-effects models [85] might provide a more appropriate method for generating these trophic niche metrics than ANOVAs, as they explicitly account for repeated sampling (e.g. sampling of multiple scute layers from individual turtles) and are robust to violations of assumptions of normality of residuals and random effects often present in ecological data generated from wild animals [85,86]. The same trophic niche metrics (WIC, BIC, TNW and WIC/TNW) were therefore calculated using linear mixed-effects models in the R package *lme4* [87] and formulated as

δ∼layer+(1|turtle)

where *δ* represents either *δ*^13^C or *δ*^15^N, *layer* represents the layer of scute within each sample (the fixed effects), and *turtle* represents individual turtles (the random effects). *δ*_SCUTE_ data were modelled against scute layers (the fixed effects) for all individual turtles (the random effects), and the intercept and slope were allowed to vary between individual turtles, which were assumed to exhibit endogenous differences in isotopic assimilation. WIC was extracted as the residual variance of the random effects, and BIC was extracted as the variance of the random effects. TNW and WIC/TNW were calculated as previously stated.

Bivariate isotopic niche metrics were calculated in a Bayesian framework using the R package *SIBER* [26]. *SIBER* uses a Bayesian implementation to calculate ellipses for groups (i.e. subpopulations) and quantifies the area of ellipses prior to posterior density calculations, (SEA), a maximum-likelihood corrected SEA (SEAc), the SEA of Bayesian ellipses (SEAb), as well as the overlap between group ellipses. SEA and all of its permutations are estimates of group isotopic niche specialization. SIBER then constructs community (i.e. population) level convex hulls based on posterior distributions from group ellipses and calculates Layman metrics [26,88] that quantify isotopic niche characteristics based on these hulls. *δ*^15^N Range and *δ*^13^C Range are the range of *δ*^15^N and *δ*^13^C values, respectively, represented by the most enriched and most depleted Bayesian ellipse centroids [26,88]. Hull area (HA) is the area of community-level convex hulls derived from Bayesian ellipse centroids fitted to groups and approximates isotopic niche breadth. Centroid dispersion (CD) is the mean distance from Bayesian ellipse centroids to convex hull centroids, and nearest neighbour distance (NND) is the mean distance between the centroids of each group and its nearest neighbour within communities [26,88]. Both CD and NND approximate group specialization relative to community isotopic niches. These metrics provide holistic quantifications of group and community isotopic niches by taking *δ*^13^C and *δ*^15^N into account simultaneously [88]. Bayesian implementations of ellipses and convex hulls use individual variation to calculate ellipses and convex hulls, which provides more accurate estimates of these metrics as well as associated error in their estimation [26]. For epidermis data, lifestages were treated as groups within one community, and SEA-values and ellipse overlap were used to compare isotopic resource use between adults and subadults. For scute data, individual turtles were treated as groups for ellipse calculations, and lifestages were treated as communities for convex hull and Layman metric calculations. Layman metrics were used to compare isotopic resource use between adults and subadults.

Variation in isotope data with individual size was examined using linear models (epidermis data) and linear mixed-effects models (scute data) to determine if and how isotopic resource use shifts with size in loggerhead turtles, and to remove error inherent in assigning lifestages based on individual size. Epidermis and scute *δ*^13^C and *δ*^15^N data met the assumptions of linear models and linear mixed-effects models, as checked in plots of the raw data and model residuals. Linear models were created for epidermis data using the base R function *lm()* and formulated as

δ∼size

where *δ* represents either *δ*^13^C or *δ*^15^N, and *size* represents CCLmin. Linear mixed-effects models were created for scute data using the function *lmer()* from the package lme4 [87] and formulated as

δ∼size+(1|turtle)

where *δ* represents either *δ*^13^C or *δ*^15^N, *size* represents CCLmin (the fixed effects), and *turtle* represents individual turtles (the random effects). Models were tested against null models formulated as

δ∼1

for linear models or

δ∼1+(1|turtle)

for linear mixed-effects models using a likelihood test in the base R function *anova()*. This served to determine if size explained significantly (*α* = 0.05) more variation in isotopic data than an arbitrary integer.

## 3. Results

**Figure 2.**
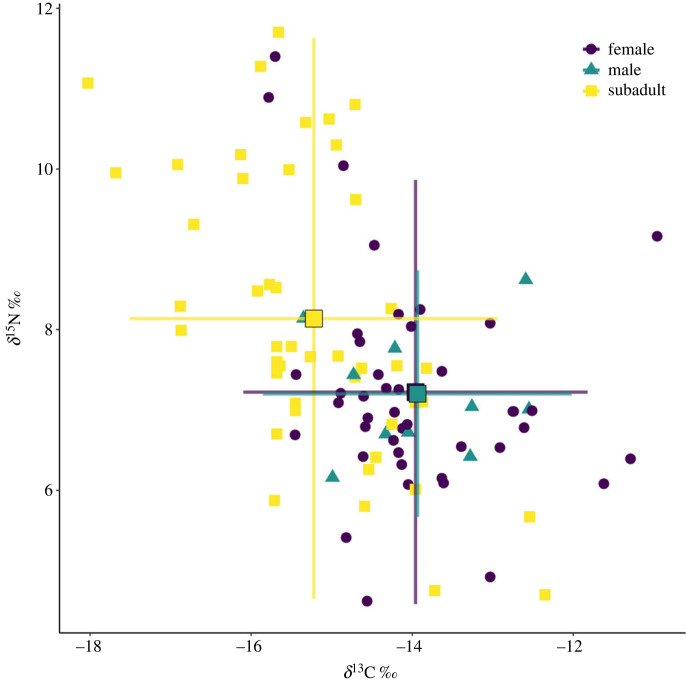
Biplot of *δ*^13^C and *δ*^15^N for epidermis samples. Colour and shape indicate sex (for individuals classified as adults) or lifestage (for subadults). Larger squares indicate group means, and bars indicate s.e. from the mean.

**Figure 3.**
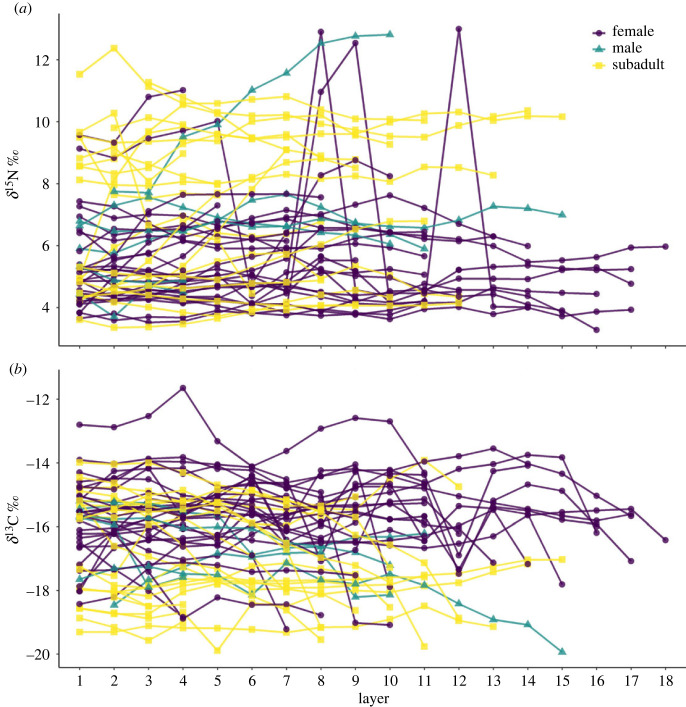
Plots of scute *δ*^15^N (*a*) and *δ*^13^C (*b*) by scute layer for individual turtles (lines). Colour and shape indicate sex (for individuals classified as adults) or lifestage (for subadults).

One hundred and thirteen loggerhead turtles were captured and sampled between 2016 and 2022 (electronic supplementary material, table S1). Capture numbers varied between months, but adults and subadults were caught in similar numbers throughout each month (electronic supplementary material, table S1). CCLmin of captured turtles ranged from 53.6 cm to 105.8 cm (mean: 80.1 ± 12.5 cm s.d.). Fifty-three turtles were classified as subadults, 60 were classified as adults, and of these 10 adult turtles were classified as males and the remaining adults were considered to be females. *δ*^13^C and *δ*^15^N were analysed for 98 epidermis and 57 scute samples (figure 2) from captured turtles. These data included samples from 53 adults (including 10 males) and 45 subadults. *δ*^15^N_EPI_ ranged from 4.62 to 11.70 ‰ (mean: 7.64 ± 1.58 ‰ s.d., electronic supplementary material, table S2) and *δ*^13^C_EPI_ ranged from −18.01 to −10.95 ‰ (mean: −14.54 ± 1.27 ‰ s.d.; figure 2; electronic supplementary material, table S2). Forty-nine central and 8 distal scute samples were analysed. Scute samples with less than four layers (*n* = 1) and duplicate samples from recaptures (*n* = 1) were excluded from analyses, leaving a total of 55 scute samples. This included 34 adults (including 6 males) and 21 subadults. Scute samples ranged from 4 to 18 layers thick (mean: 10 ± 4 layers s.d.). *δ*^15^N_SCUTE_ (figure 3*a*) ranged from 3.28 to 12.99 ‰ (mean: 6.19 ± 2.13 ‰ s.d.) and *δ*^13^C_SCUTE_ (figure 3*b*) ranged from −22.12 to −11.65 ‰ (mean: −16.20 ± 1.49 ‰ s.d.). Epidermis isotope data exhibited significantly lower *δ*^15^N and higher *δ*^13^C (Students *t*-test; *α* = 0.05) than data generated from turtles foraging in the eastern Gulf of Mexico in prior studies [80,81] and save for between *δ*^15^N from [81] and subadult loggerheads (electronic supplementary material, table S3). Isotope data from subadult loggerheads was overall more similar to those from prior studies [80,81] than were isotope data from adult loggerheads, albeit still significantly different.

Trophic niche metric estimations may be found in table 1. Metrics calculated using ANOVAs and linear mixed-effects models were orders of magnitude different in some cases, but showed the same trends within and between all turtles, adults and subadults. Adults displayed lower WIC_N_, but higher WIC_C_ than subadults. Adults exhibited lower BIC_N_ and BIC_C_, and smaller TNW_N_ and TNW_C_, than subadults. Subsequently, WIC/TNW_N_ and WIC/TNW_C_ suggested that adults exhibited slightly more generalized resource use (higher WIC/TNW) than subadults, although WIC/TNW values for both lifestages were well below expected values for true generalists [27,83,84].

**Table 1.** Trophic niche metrics for adult and subadult loggerhead turtles captured between 2016 and 2022 based on scute isotopic data. ‘ANOVA’ indicates values calculated using ANOVAs (as in [76,83,84]). ‘LMM’ indicates values calculated using linear mixed-effects models. WIC: within individual component of variation. BIC: between-individual component of variation. TNW: trophic niche width. WIC/TNW: degree of individual specialization. Subscript C: values calculated from *δ*^13^C data. Subscript N: values calculated from *δ*^15^N data.

WIC_C_	BIC_C_	TNW_C_	WIC/TNW_C_	WIC_N_	BIC_N_	TNW_N_	WIC/TNW_N_	
ANOVA								
all	0.69	16.68	17.37	0.04	1.04	36.32	37.37	0.03
adults	0.68	10.99	11.67	0.06	1.21	19.47	20.68	0.06
subadults	0.72	17.08	17.79	0.04	0.70	44.98	45.68	0.02
LMM								
all	0.83	1.32	2.16	0.39	1.03	1.93	2.96	0.35
adults	0.82	0.99	1.81	0.45	1.11	1.46	2.57	0.43
subadults	0.84	1.43	2.27	0.37	0.82	2.21	3.03	0.27

**Figure 4.**
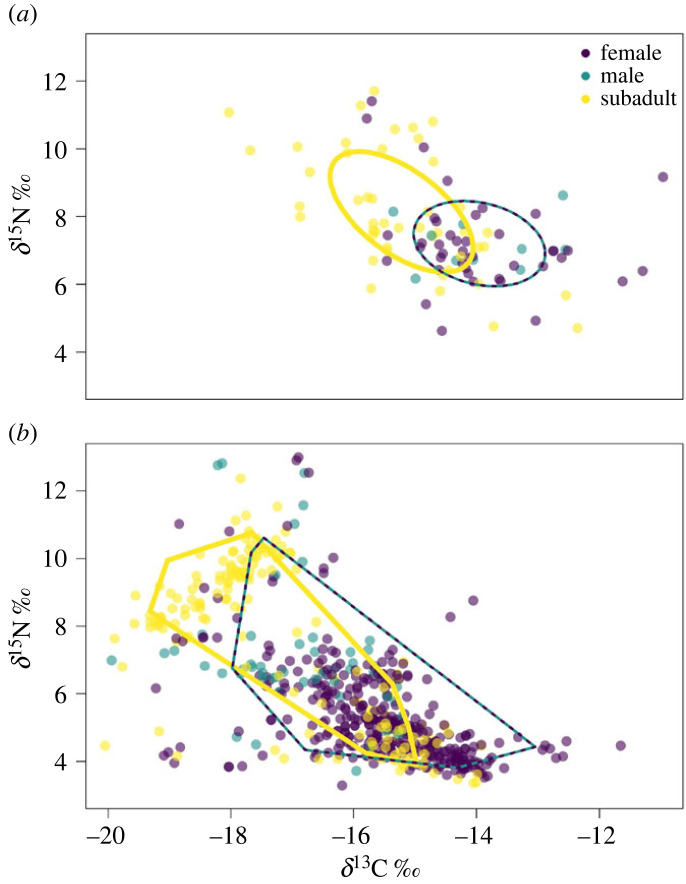
Stable isotope biplots for epidermis samples with SIBER ellipses (*a*) and scute samples with SIBER hulls (*b*). Colour indicates sex (for individuals classified as adults) or lifestage (for subadults). Adults are grouped together for analyses, hence the bi-coloured ellipse (*a*) and hull (*b*).

Bayesian ellipses constructed from epidermis data (figure 4*a*) showed that subadults (SEA = 5.24, SEAc = 5.36, SEAb = 5.21) had a larger isotopic niche than adults (SEA = 4.11, SEAc = 4.20, SEAb = 4.00), although there was substantial overlap between the two (Maximum-likelihood ellipse overlap = 19.53 ‰^2^). Layman metrics calculated from convex hulls based on scute data (figure 4*b*, table 2) indicated that these convex hulls displayed similar overall patterns to Bayesian ellipses (figure 4), but suggested that adults used a slightly larger isotopic niche (*δ*^15^N Range = 7.19 ‰, *δ*^13^C Range = 5.24 ‰, HA = 18.40 ‰^2^) than subadults (*δ*^15^N Range = 7.18 ‰, *δ*^13^C Range = 5.09 ‰, HA = 16.60 ‰^2^). Convex hulls also suggested that individual adults use less of their niche space (NND = 0.40 ‰, CD = 1.51 ‰) than individual subadults (NND = 0.58 ‰, CD = 2.50 ‰).

**Table 2.** Layman metrics for adult and subadult turtles based on scute isotopic data. HA: hull area. NND: nearest neighbour distance. SDNND: standard deviation of NND. CD: centroid dispersion.

	*δ*^15^N Range	*δ*^13^C Range	HA	NND	SDNND	CD
adults (*n* = 34)	7.15	5.24	18.40	0.40	0.35	1.51
subadults (*n* = 21)	7.18	5.09	16.60	0.58	0.36	2.50

Linear models and linear mixed-effects models demonstrated slight but significant (alpha = 0.05) isotopic variation with size in foraging loggerheads (electronic supplementary material, figure S1). Epidermis *δ*^13^C increased by 0.042 ‰ per centimetre CCLmin (electronic supplementary material, figure S1A; $\chi_{78,79}^{2}=19.6$, *p* < 0.001), but epidermis *δ*^15^N did not vary with size (electronic supplementary material, figure S1C; $\chi_{78,79}^{2}=-6.5$, *p* = 0.12). Scute *δ*^13^C increased by 0.031 ‰ per cm CCLmin (electronic supplementary material, figure S1B; $\chi_{3,4}^{2}=5.38$, *p* = 0.02), and scute *δ*^15^N decreased by 0.041 ‰ per centimetre CCLmin (electronic supplementary material, figure S1D; $\chi_{3,4}^{2}=4.41$, *p* = 0.04).

## 4. Discussion

This work generated a nuanced characterization of the ecological niche of foraging loggerhead sea turtles in the eastern Big Bend and constitutes an important contribution towards a holistic understanding of the at-sea behaviour of these imperiled, highly migratory animals. Partitioning and differences in resource use by lifestage were apparent in loggerhead turtle realized ecological niches. Subadult loggerheads generally exhibited depleted *δ*^13^C and enriched *δ*^15^N relative to adult loggerheads (figures 2–4, electronic supplementary material, S1, table S2), although there was substantial niche space overlap between subadults and adults (figures 2–4). Subadult loggerheads also exhibited more within-individual specialization and between-individual variation than adult loggerheads (tables 1 and 2) and broader univariate isotopic niches.

Comparing trophic niche metrics from adult and subadult loggerheads sampled in the eastern Big Bend to those from adult male loggerheads sampled in Florida Bay and off the Atlantic Coast of the southeastern United States [84] revealed broad differences in between-individual, within-individual and overall isotopic variation from loggerheads between these areas. This suggests either high variability in resource use between loggerheads that forage at different locations, or high variability in isotopic gradations within foraging areas. It is difficult to determine the extent to which either of these phenomenon are dominant, but future studies that generate trophic niche metrics for loggerheads and other sea turtles at important high-use areas using ANOVAs [60,83,84] or, as presented here, linear mixed-effects models, can chip away at some of this complexity. Notably, trophic niche metrics generated using ANOVAs showed the same overall trends as those generated using linear mixed-effects models, although the values were in some cases orders of magnitude different (table 1). Metrics calculated using linear mixed-effects models were more similar to the overall and within-individual isotopic ranges, and average between individual differences, seen in the raw data (figure 3), perhaps due in part to parameterization of random effects (i.e. individual turtles) in the linear mixed-effects model framework [85]. However, as the overall trends in trophic niche metrics and replicability within and between studies are of high importance, future studies should consider generating these metrics using both ANOVA and linear mixed-effects model approaches to draw informative conclusions about isotopic niches in their focal species.

Although some loggerheads captured here were within the estimated size range of new recruits to neritic environments in the northwest Atlantic Ocean (approx. 43–68 cm SCL, which should be slightly smaller than paired CCLmin; [89] and references therein), there was no evidence of offshore-to-inshore shifts (i.e. neritic recruitment) in scute isotope data (figure 3; e.g. as in [76]). Only one juvenile loggerhead (33.9 cm SCL; [65]) has been reported from the study site, and it is possible that oceanic loggerheads first recruit to other neritic areas at smaller sizes before transitioning to the study site or similar habitats (e.g. as in green turtles; [76]). Increased efforts to understand sea turtle habitat preferences, especially those displayed by juveniles, in the eastern Big Bend and more broadly the west Florida shelf [64,67] will be critical towards determining if juvenile loggerheads in this region recruit to unique, currently unmonitored areas, or if isotopic baselines disguise oceanic-neritic shifts.

Intraspecific partitioning by loggerheads at this study site may occur along both bionomic and/or scenopoetic axes, particularly regarding forage item preference and foraging locations, respectively. Most subadults may prefer higher trophic level prey items than adults. It is difficult to determine what these preferences may be, as loggerheads are opportunistic omnivores [63,90] and potential isotopic forage item data (i.e. for use in isotopic mixing models) do not exist from the study site. Prior studies of gut contents from stranded loggerheads on the Atlantic coast of the southeastern United States indicated that subadults might primarily consume fish before switching to a gastropod-dominated diet as adults [91,92]. However, existing isotopic forage item data from the Gulf of Mexico [93] suggest that most fishes' *δ*^15^N is higher (mean = 10.64 ± 2.36 ‰ s.d.) than that observed in loggerheads sampled here, and are in line with previous studies suggesting that neritic loggerheads forage on bivalves (mean = 6.51 ± 1.24 ‰ s.d.), crabs (mean = 7.04 ± 2.53 ‰ s.d.) and/or gastropods (mean = 6.49 ± 1.94 ‰ s.d.) [63,90,93]. It is also possible that subadult loggerheads consume a mixture of these items, including fishes. Given isotopic heterogeneity within the Gulf of Mexico [81,93,94] isotopic prey item data from multiple taxa in the study site (akin to those compiled in [93]) should be generated for use in isotopic mixing models to determine which forage items subadults and adults prefer at this site to better understand bionomic intraspecific niche partitioning.

Subadult and adult loggerheads may display similar bionomic (i.e. trophic), but different scenopoetic (i.e. spatial), resource use preferences. Variation in *δ*^13^C and *δ*^15^N may reflect scenopoetic partitioning in resource use between individuals and groups of individuals [25,58]. Subadult and adult loggerheads displayed differences in *δ*^13^C (figures 2 and 4; electronic supplementary material, table S2), but there was no apparent broad spatial difference between adult and subadult loggerhead capture locations within the study site (figure 1). It is possible that subadult and adult loggerheads use different habitats within the study site and eastern Big Bend region for different purposes. In the eastern Gulf of Mexico, *δ*^13^C has been measured to decrease by 0.03–0.06 ‰ per metre depth (for context, depth increases by approximately 1 m for every 5 km in the Big Bend region [95]), and *δ*^15^N has been measured to increase from east to west by 0.49–0.64 ‰ per Degree longitude and from north to south by 0.44–0.76 ‰ per Degree latitude (1 Degree = approx 111 km) in three species of fishes [96]. These patterns are potentially due to decreasing primary productivity with depth (which leads to decreased *δ*^13^C) [97–99]; spatial variability in nitrogen fixation (which leads to decreased *δ*^15^N) [100,101] and denitrification (which increases *δ*^15^N) [96]. Although isotopic data are not directly comparable between species, the biogeochemical processes that dictate baseline variation in isotopes affect tissue isotope content in all consumer species. If subadults and adults do not display strictly bionomic niche partitioning, isotopic signatures observed in subadult loggerhead tissue could therefore reflect foraging excursions to deeper, northern, or western waters relative to the study site.

Loggerheads are known to use multiple distant foraging areas within the span of just a few months [102], and adult loggerheads persistently forage at discrete sites > 10 m deep in the eastern Gulf of Mexico [103]. Isotopic data from tracked female loggerheads foraging in these areas [80,81] exhibited significantly lower *δ*^13^C_EPI_ and higher *δ*^15^N_EPI_ than almost all groups of loggerheads captured in the study site (electronic supplementary material, table S3). Loggerheads captured in this study exhibited consistency in resource use over time (table 1, figure 3), and, notably, isotopic data from some subadults captured here are more similar to those from tracked adult females than to adults captured at the study site (electronic supplementary material, tables S1 and S2). It is possible that some subadult loggerheads sampled in this study may forage in areas more isotopically similar to the turtles tracked in previous studies, which would be either deeper than, or to the north/west of, the study site. There is an isolated area with slightly increased depth and rugosity approximately 23 km northwest of the study site (as observed in [104]). There has been no documented effort to systematically observe loggerheads at this area, but they would certainly be capable of travelling here from the study site. Even so, it seems unlikely that isotopic baselines would shift enough over such a small distance to explain our results. Loggerheads (mostly subadults) have been observed resting near the Gulf Stream natural gas pipeline (30–70 m depth) and the Florida Middle Grounds Habitat Area of Particular Concern (25–50 m depth), approximately 150 km east of the study site [105]. Subadult loggerheads would have to make concerted efforts to reach these areas, but opportunistic foraging en route to or at these locations could account for isotopic differences between subadults and adults. If loggerheads display scenopoetic intraspecific partitioning, adult loggerheads and some subadults captured at the study site potentially reside and forage in the shallow (i.e. less than 10 m deep and typically less than 3 m deep), productive habitats that characterize the study site, while other subadult loggerheads captured at the study site may forage in deeper (i.e. greater than 10 m deep), less productive habitats, but spend at least some time in the shallow habitats that comprise the study site.

Bionomic partitioning (i.e. different prey item preferences) between loggerhead lifestages may be due to differing energetic demands between subadults and adults [14–16]. Subadults likely need to consume more-nutritious, and subsequently *δ*^15^N-enriched [106,107], prey items than adults to sustain growth (e.g. horseshoe crab [*Limulus polyphemus*] versus cannonball jellyfish [*Stomolophis meleagris*]) [108,109]. Adults may not need to consume particularly nutritious prey items to maintain their condition, save for prior to breeding migrations [110]. Meeting energetic demands might also pressure subadult loggerheads to specialize on specific prey items, which would explain their higher within-individual specialization in resource use relative to adult loggerheads.

Differing energetic demands, along with competition for limited resources and physiological constraints, may factor into potential scenopoetic partitioning between loggerhead lifestages. Prey items preferred by subadult loggerheads, whether or not they differ from those preferred by adult loggerheads, may exist only as patches in shallow seagrass habitats [111]. Adult loggerheads are known to display intraspecific aggression [112,113] and may actively outcompete subadults for relatively sparse forage items or locations at shallower habitats at the study site. Prey items preferred by subadult loggerheads may exist in more abundance in deeper areas where turtles have been tracked to and observed *in situ* [80,81,103]. However, foraging in deeper habitats comes with tradeoffs. Tiger (*Galeocerdo cuvier*) and bull (*Carcharhinus leucas*) sharks capable of predating turtles are more abundant in waters deeper than three meters in the Big Bend ([114]; Dean Grubbs, personal communication). Deeper waters may also be cooler than shallower waters, which could negatively impact resource assimilation [115]. Subadult loggerheads may be pressured to seek deeper forage areas, where they are at higher risk of predation and where resource assimilation is suboptimal, due to intraspecific competition for resources in shallower areas. They may even find refuge at areas with natural or anthropogenic structuring while in deeper waters [105]. Thus, subadults may make forays to deeper habitats to forage, but return to shallower habitats to minimize predation and to rest while assimilating resources [116–118].

This paradigm does not explain the substantial overlap that exists between subadult and adult ecological niches. This overlap was driven by some subadults with isotopic values more similar to adults (figures 2–4). It is unlikely that these individuals, or all of them, are competing directly with adult loggerheads and winning out, as many of these comprise the smallest subadults sampled for this study (electronic supplementary material, figure S1). These individuals may have carved out their own niche in shallow habitats at the study site by finding locations or undertaking behavioural strategies that minimize intraspecific competition, or through aggressive posturing. ‘Sneaky’ individuals are common in animal populations with observable intra- or interspecific competition. Atlantic salmon (*Salmo salar*) sneak into foraging territory occupied by larger brown trout (*Salmo trutta*) whenever the trout briefly vacate their territory [119]. Male baboons (*Papio hamadryas ursinus*) and Trinidadian guppies (*Poecilia reticulata*) use sneaky mating strategies to avoid and subvert intraspecific competition [120,121]. Conversely, more aggressive loggerheads have been shown to defend foraging patches independent of size [112], and subadult loggerheads inhabiting shallow habitats at the study site might be exhibiting aggressive posturing to defend foraging patches. Additionally, some niche overlap is likely due to opportunistic foraging outside of each lifestage's typical feeding habitat [63,90,111], if any scenopoetic partitioning is occurring.

This characterization of intraspecific niche partitioning in loggerhead turtles has direct implications for ongoing research and conservation efforts for this and other imperiled species. Analysis of isotopic data from epidermis, a tissue with short turnover time, elucidated intraspecific partitioning by size in loggerhead turtles. Analysis of isotopic data from scute, an inert tissue that allows for multiple sampling over time, strongly supported this partitioning and allowed for additional characterization of individual resource use in the light of this partitioning. When possible, future studies of ecological niches and resource partitioning in imperiled species should incorporate short- and long-term characterizations of resource use to be able to improve confidence in, and add nuance to, their characterizations. Little is known about the life-history strategies of immature turtles [64], but ecological niche characterization as conducted here has elucidated important aspects of their habitat use and behaviour. There may be comparable dividends for similar analyses of understudied demographics in other imperiled species.

Future studies should work to better characterize and understand intraspecific partitioning in loggerhead sea turtles in the Big Bend region and globally. The development of fine-scale carbon and nitrogen isoscapes and isotopic mixing models are important first steps towards assessing whether intraspecific partitioning might be bionomic or sceneopoetic in nature. If partitioning is scenopoetic, satellite telemetry could be used to monitor individual movements and spatial resource use characteristics. In the eastern Big Bend, this could be used to determine if any subadults make forays out of the study site. Identification and video monitoring of known shallow foraging habitats (i.e. specific patches) or the use of animal-borne cameras [113,122,123] could provide insight into how loggerheads interact with conspecifics in shallow habitats in the study site, and could capture any ‘sneaky’ or aggressive behaviour. Ecological niche characterization of adult loggerheads presented here strongly suggests that these individuals maintain an inshore foraging residence. Little is known in general about when and how individual adult turtles choose their foraging sites [64]. Future studies should use long-term tracking technologies (i.e. radio and/or satellite telemetry [124,125]) and genomic techniques [126] to characterize and determine the mechanism by which subadult loggerheads transition to occupying their adult ecological niches.

Knowledge of instraspecific partitioning and subsequent resource use strategies is crucial for effective conservation measures in sea turtles and other imperiled species [127,128]. Monitoring the abundance of these species at known high-use areas is important for status assessments and designating protected areas [44,45,129]. However, if some individuals are frequently moving in and out of known high-use areas, there may be the need to reassess how abundance data is being collected [129], and protected areas may need to be revised to account for expanded knowledge of habitat use. This would also be useful for protecting the habitats used by individuals when they are outside of known high-use areas. Resource use strategies that involve forays into deeper areas, for example, may expose individuals to additional threats than if they remained in one habitat [130,131], and management plans need to account for additional threats to individuals according to their resource use strategies. Similarly, threats and disturbances at known high-use areas can be managed in light of their impacts on full residents (e.g. shallower foragers), partial residents (e.g. deeper foragers) and on intraspecific competition and partitioning. Full and partial residents of high-use areas overlap in some aspects with their resource use, and the specific resources that these individuals use must be conserved. For example, prey items sought by loggerheads at the study site may reside in patches with specific characteristics (e.g. habitat type, depth, distance from shore, area, etc.), and efforts should be taken to protect these areas. Instraspecific partitioning can be driven by limited resources and further decreases to already limited resources could have drastic impacts on imperiled species [132]. For loggerheads in the study site and the broader eastern Big Bend, habitat degradation in, or extirpation from, shallow habitats (e.g. during increased anthropogenic activities; [133]) could force a majority of individuals to forage and reside in deeper habitats. If subadults are unable to rest and assimilate resources in shallow habitats, there may be immediate impacts on individual fitness and long-term impacts to population stability. Sea turtles at this site are already impacted by vessel traffic [133], and seagrass habitats in the Gulf of Mexico have undergone substantial fluctuations in abundance and quality [134,135]. Efforts to mitigate vessel interactions and protect the existing habitat, among other efforts, would be beneficial for loggerheads at this important, high-use area. Conservation efforts for sea turtles and other imperiled species can therefore use knowledge of instraspecific partitioning, resource use strategies, and existing threats to design nuanced and effective conservation plans.

## Data Availability

Isotope data available on Dryad Digital Repository: https://doi.org/10.5061/dryad.bvq83bkf6 [136]. All R Scripts for analyses and figures available on GitHub: https://github.com/FuentesLab/Silver-Gorges_et_al_2023_RSOS [137] and Zenodo: https://doi.org/10.5281/zenodo.7843440 [138]. The data are provided in the electronic supplementary material [139].
